# Detection Accuracy and Latency of Colorectal Lesions with Computer-Aided Detection System Based on Low-Bias Evaluation

**DOI:** 10.3390/diagnostics11101922

**Published:** 2021-10-17

**Authors:** Hiroaki Matsui, Shunsuke Kamba, Hideka Horiuchi, Sho Takahashi, Masako Nishikawa, Akihiro Fukuda, Aya Tonouchi, Natsumaro Kutsuna, Yuki Shimahara, Naoto Tamai, Kazuki Sumiyama

**Affiliations:** 1 Department of Endoscopy, The Jikei University School of Medicine, 3-25-8 Nishishimbashi, Minato-ku, Tokyo 105-8461, Japan; hiro24tht80@yahoo.co.jp (H.M.); kanba@jikei.ac.jp (S.K.); takeishihideka34@gmail.com (H.H.); tamai-naoto@jikei.ac.jp (N.T.); 2 Clinical Research Support Center, The Jikei University School of Medicine, 3-25-8 Nishishimbashi, Minato-ku, Tokyo 105-8461, Japan; sho@jikei.ac.jp (S.T.); mnishikawa@jikei.ac.jp (M.N.); 3 LPixel Inc., 1-6-1 Otemachi, Chiyoda-ku, Tokyo 100-0004, Japan; fukuda@lpixel.net (A.F.); tonouchi@lpixel.net (A.T.); kutsuna@lpixel.net (N.K.); shimahara@lpixel.net (Y.S.)

**Keywords:** artificial intelligence, computer-aided detection, colonoscopy, colorectal lesions, deep learning

## Abstract

We developed a computer-aided detection (CADe) system to detect and localize colorectal lesions by modifying You-Only-Look-Once version 3 (YOLO v3) and evaluated its performance in two different settings. The test dataset was obtained from 20 randomly selected patients who underwent endoscopic resection for 69 colorectal lesions at the Jikei University Hospital between June 2017 and February 2018. First, we evaluated the diagnostic performances using still images randomly and automatically extracted from video recordings of the entire endoscopic procedure at intervals of 5 s, without eliminating poor quality images. Second, the latency of lesion detection by the CADe system from the initial appearance of lesions was investigated by reviewing the videos. A total of 6531 images, including 662 images with a lesion, were studied in the image-based analysis. The AUC, sensitivity, specificity, positive predictive value, negative predictive value, and accuracy were 0.983, 94.6%, 95.2%, 68.8%, 99.4%, and 95.1%, respectively. The median time for detecting colorectal lesions measured in the lesion-based analysis was 0.67 s. In conclusion, we proved that the originally developed CADe system based on YOLO v3 could accurately and instantaneously detect colorectal lesions using the test dataset obtained from videos, mitigating operator selection biases.

## 1. Introduction

Population-based programs for screening colorectal cancer (CRC) have been widely adopted in several countries, owing to the high prevalence of CRC [1]. Colonoscopy is the most sensitive and reliable examination for detecting CRC and precursor lesions [2]. It also facilitates the immediate prophylactic removal of early neoplastic lesions. A series of cohort and case-control studies demonstrated that colonoscopy with prophylactic lesion removal could reduce the incidence and mortality of CRCs [3,4,5,6]. It is reported that most post-colonoscopy CRCs (PCCRCs) are mainly caused by lesions missed during previous colonoscopies [7]. The insufficient quality of an examination substantially increases the risk of PCCRC and mortality of CRC [8]. The adenoma detection rate (ADR) is considered the most reliable indicator of an endoscopist’s ability to detect neoplastic lesions [9]. The ADR of endoscopists is known to vary even amongst the experts, and the risk of PCCRC can increase if the examination is performed by an endoscopist with ADR < 20% [10]. Although an array of novel imaging technologies has been developed and the imaging quality of colonic lesions has been consequently improved, their effectiveness for improving the detectability of colorectal lesions is still insufficient [11,12,13].

Image recognition based on deep learning (DL) has been increasingly applied in medical imaging analysis [14,15]. Various DL-based computer-aided detection (CADe) systems for colonoscopy have been developed to aid in the detection of colorectal adenomatous lesions. Pioneering studies have demonstrated that CADe could accurately detect colorectal lesions with a sensitivity of over 90% [16,17,18,19]; however, the diagnostic performance of CADe, including sensitivity and specificity, has been evaluated retrospectively in most studies, using readily available representative still images and edited video clips stored in a medical record. This involves a substantial risk of selection bias associated with the unavoidable manipulation of the rate of the presence of objects [20,21]. In addition, the accuracies could be overrated in the retrospective settings without evaluating the latency of the detection by CADe from the initial appearance of lesions or human detection. Meanwhile, the advantage of assistance from a DL-based CADe system over non-assisted human perception has already been demonstrated in several randomized controlled trials (RCTs), including our study evaluating ADR or the adenoma miss rate (AMR) as the primary outcome. The result was confirmed in a series of meta-analyses, some of which analyzed the outcomes of RCTs [22,23,24,25]. However, RCT demands a significant amount of effort and time; retesting each updated DL-based algorithm using RCT may be suboptimal as a reference to set the direction of algorithmic developments. Therefore, it is imperative to establish a simpler and more reliable evaluation standard for the unit testing to retrospectively evaluate the diagnostic performance of CADe by mitigating the risks of selection bias [26].

In this study, we originally developed a CADe system to detect and localize colorectal lesions by modifying You-Only-Look-Once version 3 (YOLO v3). The aim of this study was primarily to assess the performance of the CADe system for colorectal lesions in fair testing settings, eliminating operator selection biases by automatically sampling a validation dataset from unedited videos of endoscopic observations without excluding low-quality images in the image-based analysis. Furthermore, the latency of the detection by CADe from the initial appearance of lesions was conducted by reviewing the videos of the entire colonic inspection in the same cohort.

## 2. Materials and Methods

### 2.1. DL-Based Algorithm to Assist Lesion Detection and Localization

The CADe system evaluated in this study was developed by modifying YOLO v3 to assist the detection and localization of colorectal lesions during a standard colonoscopy in real time. YOLO v3 is a DL-based image recognition algorithm that enables real-time object detection while ensuring a processing speed superior to other convolutional neural networks (CNNs). The developed system achieves a fast processing speed, which is partially improved using YOLO v3. The system is compatible with routinely used imaging techniques, including white light imaging (WLI), chromoendoscopy (CE), and narrow-band imaging (NBI).

We trained an object detection model based on YOLO v3, which contains several CNN architectures. The weights of the architectures were initialized through training on an ImageNet data corpus [27] before training on our data. Each architecture shared similar components—convolutional layers, activation functions, batch normalization [28], up-sampling, and skip connections [29,30]. We used the leaky rectified linear unit [31] as the activation function. The model did not contain a fully connected layer or a pooling layer; this enabled it to process images of all sizes.

In the final convolutional layer, the positions of the objects and objectness were output using logistic regression, and class predictions were output using independent logistic classifiers. Using the stochastic gradient descent optimizer, we optimized two types of losses during training—squared error loss for object positions and cross-entropy loss for objectness and class predictions. For implementation, the darknet software libraries (http://pjreddie.com/darknet/ accessed on 18 October 2018) were used. Data augmentation was applied to avoid overfitting of the training data and to generate additional data by applying random transformations to the images during training. Random mirroring, distortion, shift, and resizing were used for this purpose. Additionally, in the lesion-based analysis, to reduce false positive detection, a bounding box was set to appear on a monitor when the CADe system detects the presence of a lesion in at least 3 of any 5 sequential frames. This setting was determined via trial-and-error.

### 2.2. Objectives for Training and Validation

We trained the CADe system with stored still images and images extracted from videos recorded at Jikei University between July 2013 and March 2018. The total number of cases included in the dataset was 4080 (3544 still images, 536 from videos), which contained 26,462 lesions (24,860 still images, 1602 from videos). All images and video clips were obtained under a standard colonoscopy setting (endoscopes used: PCF-H290I, PCF-H290ZI, CF-HQ290I, CF-Q260AI, PCF-Q260AI, PCF-Q260AZI, CF-H260AZI, and PCF-Q260JI, Olympus Optical Co. Tokyo, Japan; video processors used: EVIS LUCERA CV-260 and VIS LUCERA ELITE system, Olympus, Tokyo, Japan). The CADe system was eventually trained with 84,550 images (79,079 images with lesions and 5471 without lesions).

Validation of the CADe system was retrospectively conducted by collecting and reviewing videos of the colonoscopies of patients who underwent endoscopic resection, excluding those who underwent endoscopic submucosal dissection (ESD) of colorectal lesions between June 2017 and February 2018 at the Jikei University Hospital. Cases that did not record the intubation or withdrawal of the scope as well as cases used for CADe training were excluded from the analysis. Then, we selected 20 cases from that period for evaluation using a random number table, as we assumed that 2000–4000 images would be necessary for validation in reference to preceding research, and 100–200 images could be obtained for each case. Subsequently, we evaluated the performance of the CADe system by performing two analyses—an image-based analysis to calculate the diagnostic performances using automatically sampled still images from videos, and a lesion-based analysis using video clips to measure the latency in the detection of lesions from their initial appearance. The study was approved by the ethics committee of the Jikei University School of Medicine, Tokyo, Japan (no. 30-173(9194)), and it conforms to the provisions of the Declaration of Helsinki. As a retrospective study, informed consent was obtained in the form of opting out on the website.

### 2.3. Image-Based Analysis to Calculate Diagnostic Performance Using Automatically Sampled Still Images

The still images for the validation dataset to calculate the diagnostic performance of the CADe system were automatically and blindly extracted from the video clips, including insertion to withdrawal of the scope at intervals of 5 s, without eliminating poor quality images. Then, we excluded any images with (1) the outside of the colorectum; (2) colorectal lesions and ulcers after resection; (3) imaging modes other than WLI, CE, and NBI; (4) artificial objects (endoscopic devices); (5) submucosal fluid bleb following needle injection; (6) more than two colorectal lesions.

#### 2.3.1. Receiver Operating Characteristic (ROC) Curve Drawing to Determine the Optimal Threshold

An expert endoscopist reviewed the still images, and labeled lesions contained in the images were framed with a rectangular bounding box (ground truth bounding box).

The CADe system separately analyzed the still images, and bounded boxes (predicted bounding boxes) were drawn around detected lesions in various probability thresholds.

Thereafter, the still image dataset with ground truth bounding boxes and CADe-predicted bounding boxes were compared for calculating sensitivity and specificity in various probability thresholds based on the definitions of true positive (TP), true negative (TN), false positive (FP), and false negative (FN), as described in Section 2.3.2. ROC curves were plotted for each imaging modality (Overall, WLI, CE, or NBI) by varying the probability thresholds from 0.01 to 0.99 at intervals of 0.01. We defined the optimal cutoff value of the probability threshold for each modality when it was at the maximum Youden index. [32].

#### 2.3.2. Definitions of True Positive, True Negative, False Positive, and False Negative

**Figure 1:**
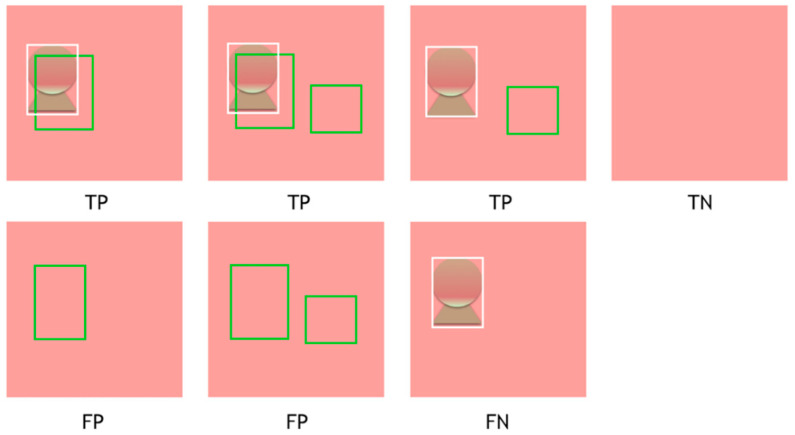
Schematic presentation of the definition of TP, TN, FP, and FN in the image-based analysis. White bounding box: ground truth bounding box provided by expert endoscopist. Green bounding box: predicted bounding box provided by CADe. TP, true positive; TN, true negative; FP, false positive.

**Figure 2:**
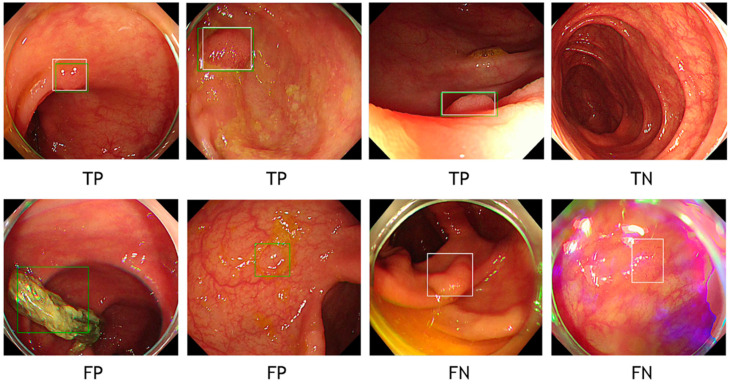
Ground truth and predicted bounding boxes overlaid on endoscopic images. White box: ground truth bounding box provided by expert endoscopist. Green box: predicted bounding box provided by CADe.

In the image-based analysis, we primarily evaluated if the CADe system could demonstrate lesion-positive frames with bounding boxes in order to calculate the diagnostic performances, that is, AUC, sensitivity, specificity, PPV, NPV, and accuracy as binary variables in a 2 × 2 table. Therefore, TP, TN, FP, and FN in this study were defined as follows: Cases in which both ground truth bounding boxes and the CADe bounding boxes were in the same image were defined as TP, regardless of their overlapping. Cases without either ground truth bounding boxes or the CADe bounding boxes were solely defined as TN. When the CADe bounding boxes existed in an image without ground truth bounding boxes, the case was categorized as FP, regardless of the number of the CADe bounding boxes. Cases were defined as FN when the CADe system failed to place bounding boxes on an image with ground truth bounding boxes (Figure 1 and Figure 2.).

Subsequently, sensitivity, specificity, PPV, NPV, and accuracy were calculated using the following expressions: Sensitivity = n(TP)/(n(TP) + n(FN))

Specificity = n(TN)/(n(TN) + n(FP))

Accuracy = (n(TP) + n(TN))/(n(TP) + n(FN) + n(FP) + n(TN))

PPV = n(TP)/(n(TP) + n(FP))

NPV = n(TN)/(n(TN) + n(FN)),

where n(TP), n(FN), n(TN), and n(FP) denote the number of images of TP, FN, TN, and FP, respectively.

#### 2.3.3. Localization Accuracy for Predicted Bounding Boxes Using Intersection over Union (IoU)

Intersection over union (IoU) (the area of overlap between the predicted bounding box and the ground truth bounding box) was evaluated for CADe-predicted bounding boxes overlapping with ground truth bounding boxes. In order to identify if the CADe system could accurately predict the localization of lesions or opportunistically place bonding boxes on images with the ground truth bounding boxes, the IoU > 50 ratio for the overlapping boxes was analyzed.

### 2.4. Lesion-Based Analysis by Reviewing Videos for Evaluating the Latency of CADe

Initially, an expert endoscopist identified and defined the initial appearance of all 69 lesions clinically requiring resection by meticulously reviewing the videos during the scope withdrawal from the scene of the cecal bottom or the anastomotic site with the ileum to the scene of the anal canal, without CADe assistance for all 20 cases. The median length of the videos reviewed was 1589 s (751–3592 s). Then, the CADe system evaluated clipped video-recorded scenes around the initial lesion appearance with a median length of 15 s (7–48 s). The lesion detection time of the CADe system was assessed for each lesion by counting frames between the frame with the initial appearance of a lesion and the time when the same lesion was initially detected by the CADe system. When a lesion had not been detected by the CADe system for 5 s from the initial appearance, we considered the lesion detection by the CADe system as failed.

### 2.5. Statistical Analysis

Medians with range are presented for continuous variables. Frequencies and proportions are provided for categorical variables. The sensitivity, specificity, PPV, NPV, and accuracy of the CADe system for lesion detection were calculated using the formulas given in Section 2.3.2, and the corresponding two-sided 95% exact confidence intervals (CIs) based on the binomial distribution were estimated. The ROC curve of the CADe system was plotted, and the AUC was calculated using the trapezoidal rule, and the corresponding two-sided 95% exact CI based on the binomial distribution was estimated. The Youden index for each probability threshold of the system was calculated, and the value with the maximum Youden index was defined as the cutoff value. All statistical analyses were performed using Stata 14.2 (StataCorp LP, College Station, TX, USA).

## 3. Results

**Figure 3:**
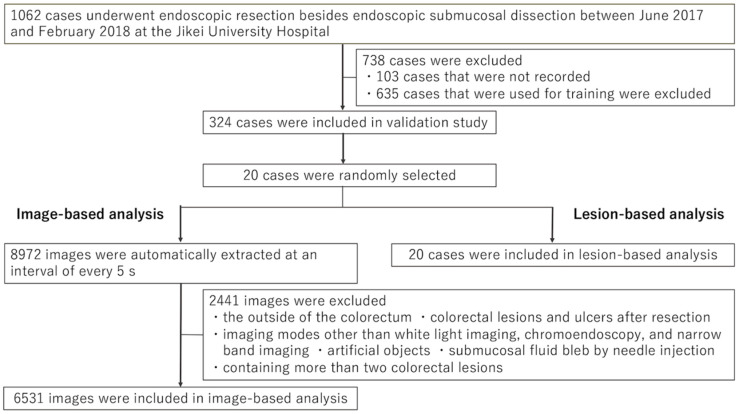
Flowchart for case enrollment and image selection for validation.

A total of 1062 cases underwent endoscopic resection besides endoscopic submucosal resection between June 2017 and February 2018 at the Jikei University Hospital (Figure 3). A total of 738 cases (103 not recorded, 635 used for training) were excluded. From the remaining 324 cases, 20 cases (16 males and 4 females, median age of 63 (36–80) and one case after a right side hemicolectomy) were randomly selected and analyzed.

### 3.1. Image-Based Analysis Using Automatically Sampled Still Images

**Figure 4:**
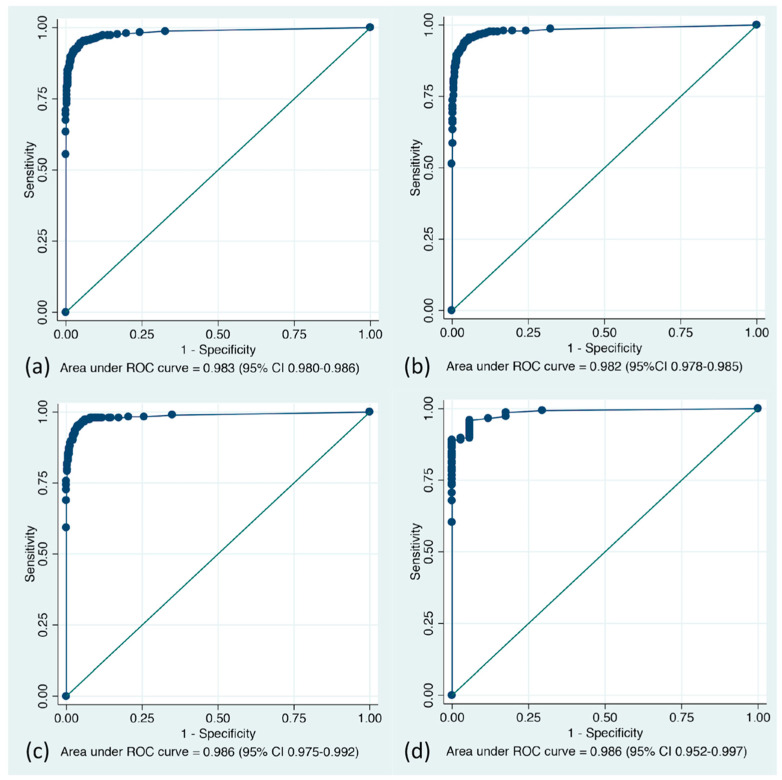
Receiver operating characteristic curves of the CADe system for overall, white light imaging, chromoendoscopy, and narrow-band imaging images in the image-based analysis. (**a**) Overall (n = 6531); (**b**) White light imaging (n = 5527); (**c**) Chromoendoscopy (n = 824); (**d**) Narrow band imaging (n = 180); ROC, Receiver operating characteristic.

Overall, videos were recorded for 20 cases for a total length of 12 h 28 min 9 s, and the number of still images extracted was 8972. The total number of images analyzed was 6531, after excluding 2441 images according to the exclusion criteria (Figure 3). The number of images with ground truth bounding boxes was 662 (662/6531, 10.1%). The analyzed images contained 5527 WLI images, 824 CE images, and 180 NBI images. The AUC of lesion detection with the CADe system was 0.983 overall, 0.982 in WLI, 0.986 in CE, and 0.986 in NBI (Figure 4). The probability thresholds regarding the maximum Youden index for overall, WLI, CE, and NBI were 0.27, 0.22, 0.43, and 0.05–0.06, respectively (Table 1). When probability thresholds of 0.27 were applied, the sensitivity, specificity, positive predictive value, negative predictive value, and accuracy were 94.6%, 95.2%, 68.8%, 99.4%, and 95.1%, respectively.

The IoU > 50 ratio for the overlapping boxes was 97.3%. The results indicate that the CADe system could accurately predict the localization of detected lesions.

### 3.2. Lesion-Based Analysis with Video Reviewing

A total of 69 lesions were endoscopically removed in 20 cases. Of those, 57 lesions were morphologically classified as elevated lesions and 12 were classified as flat lesions (morphology of lesions in the Paris classification: 1 lesion of 0-Ip, 56 lesions of 0-Is, and 12 flat lesions (0-IIa)). The median size of the lesions was 5 (2–20) mm (Table S1). The CADe system found 98.6% of the treated lesions. One diminutive adenomatous lesion that was 3 mm in size was not identified (Case #14, Lesion #2, Table S1). The median time between the initial appearance of the lesion and the CADe detection was 0.67 s (0.13–4.53) (Video S1).

## 4. Discussion

Preceding retrospective studies on DL-based CADe for colorectal lesions have shown excellent diagnostic performances with a sensitivity of 90–97.3% and specificity of 63.3–99% [16,17,18,19]. However, the study designs greatly varied, and the terms “sensitivity” and “specificity” used in these studies may be misleading, implying that they were used as statistical measures of a binary classification test on a fixed dataset, as is widely used in studies on endoscopic differential diagnosis. They could be optimal for demonstrating the diagnostic performance of CADe in the analysis of stored still images because it is clinically acceptable to perform the characterization and differentiation of lesions by freezing the endoscopic motion and selecting the best quality images with a latency time. Meanwhile, the analysis and feedback of CADe need to be performed in real time to enable endoscopists to make timely decisions. Therefore, we believe that the binary classification test of selected stored datasets would be suboptimal to evaluate the diagnostic performance of CADe. Theoretically, the diagnostic performance of CADe could be analyzed per image, per lesion, per boundary box, or per pixel at any chronological phase during a colonoscopy. Different procedures were adopted in previous studies; some studies evaluated specificity using images that did not contain a lesion separately from the analysis of sensitivity with images of lesions. The negative clinical impact of false positives by CADe with low PPV tends to be underestimated in cases where sensitivity and specificity cannot be addressed equally. In this study, we primarily evaluated whether CADe could demonstrate lesion-positive frames configuring bounding boxes to calculate the diagnostic performances as binary variables in a 2 × 2 table. The trade-off between sensitivity and specificity calculated in a 2 × 2 table enabled an ROC curve to be plotted, and it allowed us to set the reasonable cutoff value of the probability threshold for each imaging mode at the Youden index. Even though the validation dataset used in this study was blindly sampled from video clips of the entire colonoscopy procedure, including poor quality images obtained during scope intubation, the CADe system still achieved an excellent AUC (0.983 in overall, 0.982 in WLI, 0.986 in CE, and 0.986 in NBI) and desirable diagnostic performances for every imaging mode. In addition, we found that the comparative diagnostic performances could be achieved with the same cutoff value of the probability threshold set with the ROC curve in overall images regardless of the imaging mode. It warranted the usage of the cutoff value of 0.27 for the lesion-based analysis using videos. However, there was a discrepancy between WLI and other imaging modes in the PPV. We surmised that it might be explained by the difference in the situations under which the imaging modes were used. CE and NBI were mostly used during the withdrawal of the endoscope and more often after the initial detection of the suspicious area with WLI. The results imply that the PPV could be a highly sensitive reference for further improving CADe, although the impact of the prevalence of the test data needs to be considered [33]. The definitions of TP and FP for the binary data analysis made it impossible to assess if the bounding boxes predicted by CADe were overlapped on lesions, but the low NPV and high localization accuracy demonstrated using the IoU analysis indicated that the CADe system could point out the location of the polyp.

As far as we know, there is no previous study measuring the latency from the initial appearance of lesions, as we conducted in this study. Hassan et al. evaluated the time gap in detecting colorectal lesions between CADe and endoscopists and demonstrated CADe detected lesions faster than endoscopists by an average of 1.27 s [34]. In this study, the latency of the detection by the CADe system from the initial appearance of lesions had a median of only 0.67 s, although the measures taken to decrease false positives required the analysis of five sequential frames to create each bounding box. The results prove that the CADe system would detect lesions much faster than humans.

There are several limitations to this study. The diagnostic performance in this study was only tested retrospectively in a small number of therapeutic cases with a high prevalence of the disease; screening and surveillance performance should be evaluated using a testing dataset comprising a different number of lesions. It was also difficult to evaluate whether the CADe system could find more lesions than those identified by the endoscopist in this retrospective study. However, the validation data automatically sampled from the whole endoscopic inspection images during the scope intubation consequently contained a lot of low-quality images, and we confirmed that the CADe system could detect lesions even in difficult locations in the still image analysis and immediately after the appearance in the movie analysis. We recognize the need for a prospective controlled study to clinically analyze validated quality indicators to demonstrate the advantage of CADe over non-assisted human diagnosis. Meanwhile, the aim of this study was purely to conduct performance testing for the CADe system by using readily available stored videos of colonoscopies, analyzing outcomes, and mitigating operator selection biases. We believe that such validation methods would be more preferable in a developmental phase to decide the appropriate direction of research and also to reveal the strengths and weaknesses of CADe. In fact, a prospective multi-center randomized trial evaluating AMR as the primary outcome in a tandem study design, in which the CADe-assisted group achieved better AMR than the non-assisted group, was warranted from the desirable results of this retrospective trial [23].

In conclusion, the developed CADe system for assisting in lesion detection and localization achieved high sensitivity and specificity in fair testing settings, mitigating operator selection biases by automatically sampling a test data set from unedited videos of endoscopic observations, without eliminating low-quality images. Further, by reviewing the video clips of lesions, starting with the initial appearance during the colonoscopic inspection, it was demonstrated that the CADe system could detect most lesions that required removal in real time. Although the advantage of CADe should be validated in direct comparison with a human analyzing clinically approved quality indicators of the examination, such as ADR and AMR, we believe that the retrospective study under the strictly controlled environment, mitigating biases generally associated with research for computer-aided diagnosis, is valuable and should be refocused in this rapidly progressing research field.

## Figures and Tables

**Table 1:** Diagnostic performance of computer-aided detection system in image-based analysis with automatically sampled still images.

Type of Imaging Mode (No. of Lesions/No. of Images (Prevalence (%))	Youden Index	Probability Threshold	Sensitivity (95% CI)(%)	Specificity (95% CI)(%)	PPV (95% CI)(%)	NPV (95% CI)(%)	Accuracy (95% CI)(%)	IoU ≥ 0.5 (TP)(%)
Overall (662/6531 (10.1%))	0.897	0.27	94.6 (92.6–96.2)	95.2 (94.6–95.7)	68.8 (65.7–71.8)	99.4 (99.1-99.6)	95.1 (94.5–95.6)	97.3 (609/626)
WLI (334/5527 (6.0%))	0.9	0.22	95.5 (92.7–97.5)	94.5 (93.8–95.1)	52.6 (48.5–56.6)	99.7 (99.5–99.8)	94.5 (93.9–95.1)	96.9 (309/319)
	0.899	0.27	94.6 (91.6–96.8)	95.3 (94.7–95.8)	56.3 (52.1–60.5)	99.6 (99.4–99.8)	95.2 (94.6–95.8)	97.2 (307/316)
CE (182/824 (22.1%))	0.912	0.43	95.1 (90.8–97.7)	96.1 (94.3–97.5)	87.4 (81.9–91.7)	98.6 (97.3–99.3)	95.9 (94.3–97.1)	97.1 (168/173)
	0.909	0.27	96.7 (93.0–98.8)	94.2 (92.1–95.9)	82.6 (76.9–87.5)	99.0 (97.9–99.6)	94.8 (93.0–96.2)	97.2 (307/316)
NBI (146/180 (81.1%))	0.956	0.05-0.06	95.9 (91.3–98.5)	94.1 (80.3–99.3)	98.6 (95.0–99.8)	84.2 (68.7–94.0)	95.6 (91.4–98.1)	96.4 (135/140)
	0.859	0.27	91.8 (86.1–95.7)	94.1 (80.3–99.3)	98.5 (94.8–99.8)	72.7 (57.2–85.0)	92.2 (87.3–95.7)	97.0 (130/134)

PPV, positive predictive value; NPV, negative predictive value; CI, confidence interval; TP, true positive; IoU, intersection over union; WLI, white light imaging; CE, chromoendoscopy; NBI, narrow band imaging.

## Data Availability

The data that support the findings of this study are available from the corresponding author upon reasonable request.

## Supplementary Materials

The following are available online at https://www.mdpi.com/article/10.3390/diagnostics11101922/s1, Table S1: Latency of lesion detection using the computer-aided detection system in lesion-based analysis, Video S1: Colorectal lesion detection by the computer-aided detection system using video clips.
