# Supplementary material for: Detection Accuracy and Latency of Colorectal Lesions with Computer-Aided Detection System Based on Low-Bias Evaluation
Source: Diagnostics (Basel). 2021 Oct 17;11(10):1922. doi: 10.3390/diagnostics11101922 (PMC8534444; doi:10.3390/diagnostics11101922)
Supplement: Supplementary file 1 [file diagnostics-11-01922-s001.zip › diagnostics-1352305-SI/Diagnostics/Supplementary Materials Table S1 .pdf]

Supplementary Materials

Table S1

Latency of lesion detection using the computer-aided detection system in lesion-based analysis

Case No.	Polyp No.	Morphology	Size (mm)	Pathological diagnosis	Detection time (sec)
**1**	1	0-Is	3	Adenoma	1.6
**1**	2	0-Is	4	Adenoma	2.13
**1**	3	0-Is	5	Adenoma	1.93
**1**	4	0-Is	5	Adenoma	1.63
**1**	5	0-Is	10	Adenoma	0.5
**2**	1	0-Is	6	Adenoma	0.3
**2**	2	0-Is	5	Adenoma	1.53
**3**	1	0-Ⅱa	12	Adenoma	1.43
**3**	2	0-Is	3	Adenoma	2.67
**4**	1	0-Is	10	Adenoma	0.3
**5**	1	0-Is	6	Adenoma	0.13
**5**	2	0-Is	6	Adenoma	0.27
**6**	1	0-Is	3	Adenoma	0.4
**6**	2	0-Is	4	Adenoma	1.43
**6**	3	0-Is	2	Adenoma	0.9
**6**	4	0-Is	5	Adenoma	0.23
**6**	5	0-Is	6	Adenoma	0.67
**7**	1	0-Is	5	Adenoma	0.27
**7**	2	0-Is	4	Adenoma	1.67

**7**	3	0-Is	4	Adenoma	0.6
**8**	1	0-Ⅱa	2	Adenoma	0.83
**8**	2	0-Ⅱa	8	Adenoma	0.33
**9**	1	0-Is	5	Adenoma	0.67
**9**	2	0-Ⅱa	5	Hyperplastic polyp	0.57
**9**	3	0-Is	5	Adenoma	1.63
**9**	4	0-Ⅱa	5	Adenoma	0.17
**10**	1	0-Is	6	Adenoma	1.93
**10**	2	0-Is	20	Adenoma	4.53
**10**	3	0-Is	15	Adenoma	0.2
**10**	4	0-Is	3	Adenoma	0.43
**10**	5	0-Ip	12	Adenoma	0.2
**10**	6	0-Is	3	Adenoma	0.2
**10**	7	0-Is	5	Adenoma	0.37
**10**	8	0-Is	15	Adenoma	1.17
**11**	1	0-Is	3	Adenoma	1.67
**11**	2	0-Is	2	Adenoma	1.1
**11**	3	0-Is	7	Adenoma	0.83
**11**	4	0-Is	6	Hyperplastic polyp	1.1
**11**	5	0-Is	4	Adenoma	0.37
**11**	6	0-Is	4	Hyperplastic polyp	0.2
**12**	1	0-Ⅱa	8	Adenoma	3.4
**13**	1	0-Is	6	Adenoma	1.53
**13**	2	0-Is	5	Adenoma	3.43
**14**	1	0-Ⅱa	5	Adenoma	0.53

**14**	2	0-Is	3	Adenoma	Not analyzed
**14**	3	0-Ⅱa	4	Adenoma	0.6
**14**	4	0-Ⅱa	5	Adenoma	2.37
**14**	5	0-Is	3	Adenoma	2.27
**14**	6	0-Is	4	Adenoma	0.2
**14**	7	0-Is	6	Adenoma	0.67
**14**	8	0-Is	8	Adenoma	1.87
**15**	1	0-Is	2	Adenoma	1.5
**15**	2	0-Is	5	Adenoma	0.6
**15**	3	0-Ⅱa	3	Hyperplastic polyp	3.67
**15**	4	0-Is	6	Adenoma	4.33
**15**	5	0-Is	2	Adenoma	0.17
**16**	1	0-Is	5	Adenoma	1.6
**17**	1	0-Is	3	Adenoma	0.67
**17**	2	0-Is	7	Adenoma	0.17
**18**	1	0-Ⅱa	2	Adenoma	1.93
**18**	2	0-Ⅱa	6	Adenoma	0.53
**18**	3	0-Is	2	Adenoma	0.53
**19**	1	0-Is	7	Adenoma	0.13
**19**	2	0-Is	4	Adenoma	0.57
**19**	3	0-Is	3	Adenoma	0.77
**19**	4	0-Is	4	Adenoma	0.27
**19**	5	0-Is	5	Adenoma	2.63
**20**	1	0-Is	3	Adenoma	0.43

**20**	2	0-Is	3	Hyperplastic polyp	0.9
